# Evidence-based recommendations for diagnosing and managing dentine hypersensitivity in clinical practice: insights from the Middle East and Africa

**DOI:** 10.3389/froh.2025.1663984

**Published:** 2025-11-07

**Authors:** Ali Cekici, Amynah Shaikh, Arwa Alsayed, Aslan Yasar Gokbuget, Ebrahim Patel, Hesham Marei, Manal Awad, Ninette Banday, Nour A. Habib, Samira M. Osailan, Tim Theuri, Tope Emmanuel Adeyemi, Charlie R. Parkinson, Ahmed Hamdy, Juhi Thomas

**Affiliations:** 1 Periodontology Department, Faculty of Dentistry, Istanbul University, Istanbul, Türkiye; 2 Department of Oral Biology, College of Dentistry, Dow International Dental College, Karachi, Pakistan; 3 Dar Al Uloom University, Riyadh, Saudi Arabia; 4 Prosthodontis & Orthodontist & GP (PGG) Dental Clinic, Istanbul, Türkiye; 5 Wits Oral Health Centre/School of Oral Health Sciences, Faculty of Health Sciences, Gauteng Department of Health, University of the Witwatersrand, Johannesburg, South Africa; 6 Oral and Maxillofacial Surgery, College of Dentistry, Gulf Medical University, UAE Gulf Medical University, Ajman, United Arab Emirates; 7 Department of Orthodontics, Pediatric and Community Dentistry, College of Dental Medicine, University of Sharjah, Sharjah, United Arab Emirates; 8 Marigold Dental and Orthodontic Clinic, Abu Dhabi, United Arab Emirates; 9 Biomaterials Department, Faculty of Dentistry, Cairo University, Cairo, Egypt; 10 Faculty of Dentistry, Oral and Maxillofacial Surgery Department, King Abdulaziz University, Jeddah, Saudi Arabia; 11 Kenya Dental Association, Nairobi, Kenya; 12 Department of Child Dental Health, Faculty of Dentistry, Bayero University, Kano/Aminu Kano Teaching Hospital, Kano, Nigeria; 13 Haleon, Weybridge, United Kingdom; 14 Medical Affairs, Haleon, Dubai, United Arab Emirates

**Keywords:** dentine hypersensitivity, dentine hypersensitivity experience questionnaire (DHEQ), Middle East and Africa (MEA) region, tooth sensitivity, diagnostic methods, clinical management

## Abstract

Dentine hypersensitivity (DH) is a common yet often overlooked oral condition that causes pain and discomfort, negatively impacting quality of life. The prevalence of DH in the Middle East and Africa (MEA) region is relatively higher than in European countries, highlighting the need for interventions to reduce the disease burden associated with DH. A systematic approach and a thorough understanding of the condition are required for proper diagnosis and management. However, the lack of specific treatment guidelines in the MEA region poses a challenge for clinicians in identifying and managing DH. To address this, an advisory board panel of 12 dental experts from 8 MEA countries developed these consensus recommendations for DH diagnosis and management. This paper presents an overview of the clinical presentation, diagnostic challenges, and management strategies specific to the MEA region. It provides evidence-based recommendations and a simplified algorithm to guide clinicians in diagnosing and managing DH effectively. The panel underscored the importance of early diagnosis, preventive education, behavioral modification, and personalized treatment interventions, including self-care home-based therapies, for optimal DH management. Additionally, the panel emphasized the need for heightened public awareness and the integration of DH education into dental professional curricula.

## 1 Introduction

Dentine hypersensitivity (DH) is one of the most common discomforting oral conditions that causes pain (1). It affects quality of life (QoL) of patients and if not addressed, it can become a long term problem, with symptoms potentially worsening over time and negatively impacting daily activities such as speaking, eating, drinking, and toothbrushing (1–4).

Studies indicate that DH is relatively common in the Middle East and Africa (MEA) region (1, 5–7). For instance, a large survey of dental patients across several Arab countries found that roughly one-third of individuals had experienced DH, with prevalence as high as ∼13.9% in Saudi Arabia and ∼15% in Oman (7). These rates exceed many published figures from European or Asian populations (1, 8–11). In fact, global DH prevalence estimates are highly variable, a recent meta-analysis reported values ranging from about 1.3% to 92.1% (mean ≈33%), reflecting major differences in study methods, populations, and diagnostic criteria (1). Notably, most prevalence studies have been conducted outside the MEA region (8–11), and few rigorous surveys exist locally (6, 7). Since DH symptoms often go unreported by patients and may not be specifically assessed by clinicians, many cases likely go undocumented. Thus, even the relatively high prevalence reported for the MEA probably underestimate the true burden of DH in these countries (12). In other words, the real prevalence of DH in MEA is likely higher than current estimates imply, highlighting the need for targeted interventions and further epidemiological research. Diagnosing DH requires a systematic clinical approach to rule out other sources of dental pain (such as caries or pulpitis) (13). However, the MEA region generally lacks standardized, region-specific guidelines or training for DH, making its identification and management more challenging (14). In routine practice, this has led DH being frequently under-recognized (12, 14). Improving clinician awareness and establishing clear diagnostic protocols are therefore critical steps to uncovering the true extent of DH in MEA region (14).

This paper aims to provide evidence-based recommendations for the diagnosis and clinical management of DH in the MEA region. These recommendations are intended to help oral healthcare professionals (dentists and dental hygienists) and policymakers, offering a foundation for informed, evidence-based decisions. While they are not intended to establish a mandatory standard of care, these recommendations provide valuable insights to enhance patient care and support oral health practitioners.

## 2 Panel composition

On June 29th, 2024, an advisory board meeting was convened in Dubai through funding and support provided by Haleon. The panel comprised 12 distinguished dental professionals from 8 countries across the MEA region (Supplementary Table 1). Panelists represented a wide range of dental specialties, including periodontics and implantology, restorative dentistry, orthodontics, pediatric and community dentistry, and oral and maxillofacial surgery. Members were selected based on their clinical expertise, academic contributions, and representation across various dental specialties, ensuring a multidisciplinary approach throughout the discussion.

The meeting aimed to address the challenges in diagnosing and managing DH in the MEA region. Panelists engaged in structured deliberations focused on the clinical presentation of DH, diagnostic barriers, and available management strategies relevant to the regional context. Their discussions were guided by a thorough evaluation of existing international guidelines, a comprehensive literature review, and their collective clinical experiences.

The outcome of this collaboration is a set of practical, evidence-informed recommendations tailored to the MEA region. These include simplified diagnostic and management algorithms to support clinicians in delivering personalized care for DH patients. The recommendations presented in this review were developed through a structured process involving expert discussions and a comprehensive review of the literature. To ensure objectivity and scientific rigor, the draft recommendations were independently reviewed by all participating experts, including those unaffiliated with the sponsor. Each expert provided feedback, and the draft was revised accordingly to reflect a balanced and evidence-based consensus. To ensure transparency and inclusivity, all the dissenting opinions and minority viewpoints were documented and were considered in the final consensus.

## 3 Literature search

An electronic search was conducted on PubMed, Google Scholar, and ProQuest without time restrictions, using combinations of the following basic terms and Medical Subject Headings (MeSH) keywords: “Dentine hypersensitivity”, “Dentine sensitivity”, “Tooth sensitivity”, “tooth hypersensitivity”, “root sensitivity”, teeth, tooth, dental, enamel, “dentin*”. The search was restricted to clinical studies, reviews, meta-analyses, or any existing guidelines published in English. The inclusion criteria encompassed studies involving adult patients diagnosed with DH, focusing on prevalence, etiological factors, clinical features, underlying mechanism, and diagnostic barriers. We also considered studies exploring the screening, diagnosis, treatment strategies and awareness efforts among dental professionals and the public. The exclusion criteria eliminated studies involving dental pain from other causes unrelated to DH. The search and selection process of articles is presented as Supplementary Figure 1. The potential articles were reviewed to gather information and to inform the panel on the latest relevant findings on DH and served to underpin the expert opinions. Expert opinions or recommendations were graded from A to C based on the level of associated evidence, or as a good practice point (GPP), based on the grading method adapted from National Collaborating Centre for Mental Health (Supplementary Table 2) (15).

## 4 Discussion

### 4.1 Dentine hypersensitivity remains an underdiagnosed condition with a prevalence that varies across different MEA regions

#### 4.1.1 Prevalence

Identifying the precise proportion of the population suffering from DH posed a challenge during the literature search. Zeola et al. conducted a meta-analysis that estimated a global prevalence ranging widely from 1.3% to 92.1% (1). This broad range was attributed to variations in study characteristics, participant age, study size, and recruitment method (1). Most of the included studies focused on Europe (40%) and Asia (38%), followed by America (13%), Africa (6%), and Oceania (3%) (1). On average, DH affects about 33% of adults globally, highlighting its significance as a widespread clinical issue (1).

**Figure 1:**
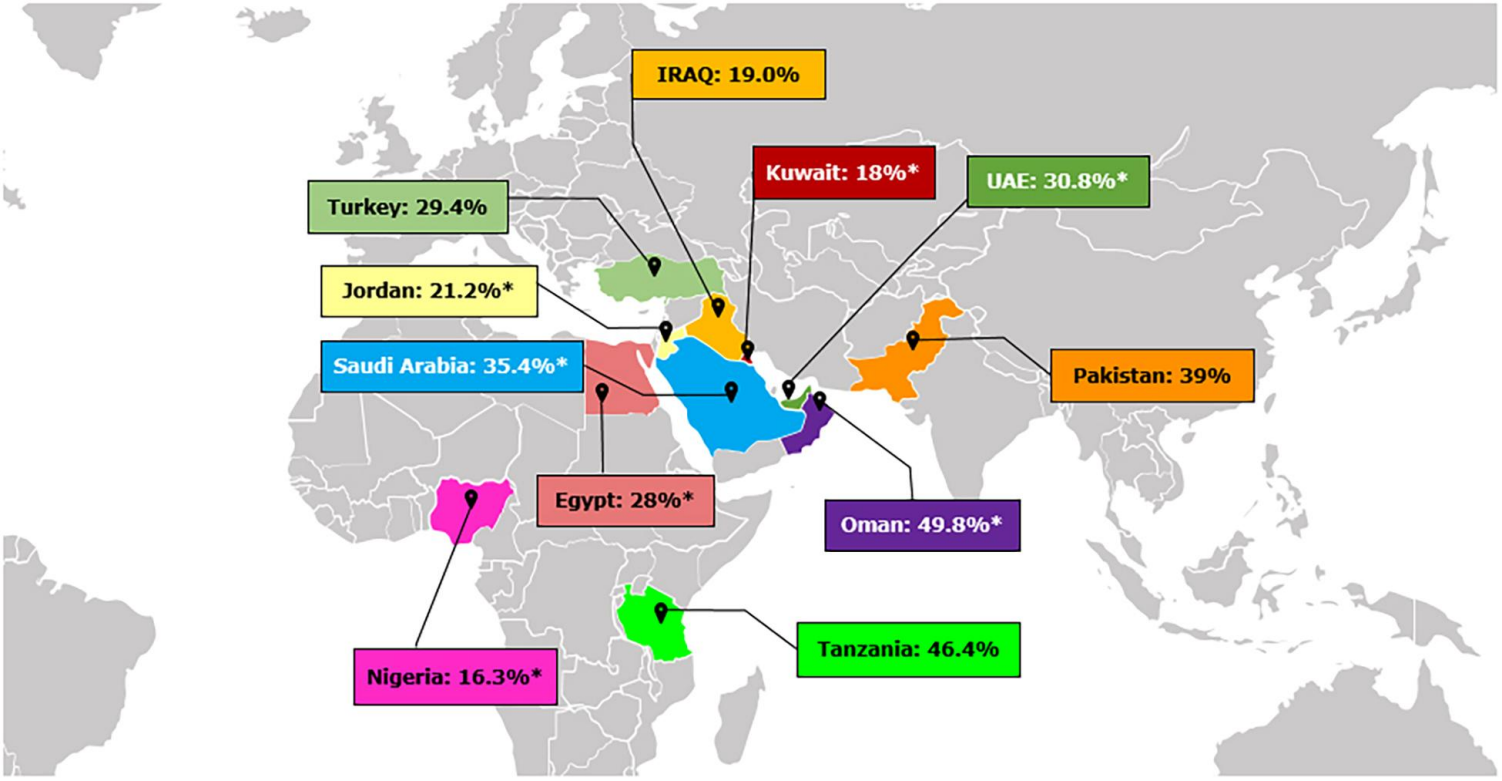
Prevalence of dentine hypersensitivity in different countries across Middle East and Africa (7, 16–20). *Indicates prevalence of DH measured by Schiff Scale and reflects percent people with Schiff 2 and 3.

Studies across the MEA region are scarce, with DH prevalence ranging from 16.3% in Nigeria to 35.4% in Saudi Arabia and 49.8% in Oman (Figure 1) (7, 16–20). Variations in socio-economic status, lifestyle factors, dietary habits, oral hygiene practices, and access to healthcare services likely explain these differences. Limited awareness of proper oral health practices and inadequate healthcare infrastructure in these countries may also contribute to the observed disparities (21).

Notably, DH shows a higher prevalence in women (5, 22) and tends to peak between 30 and 40 years of age (5). Older adults often experience reduced sensitivity, possibly due to changes in biomedical factors e.g., the development of secondary dentine and tubular sclerosis (5).

#### 4.1.2 Etiological or predisposing factors

DH primarily develops from dentine exposure due to the loss of enamel or cementum, particularly in cases of gingival recession. This exposure opens the dentinal tubules to both the oral cavity and the pulp, leading to sensitivity (2, 5). The panel suggested the importance of classifying the etiology of DH for accurate diagnosis and effective treatment. They classified these etiologies into two main categories: tooth-related and soft tissue-related causes (Table 1) (2, 5, 23). Tooth related causes include tooth wear, orthodontic treatment, aggressive tooth brushing (2, 5, 23). Soft tissue-related causes encompass gingival recession, and periodontal disease (2, 5, 23). Several factors can predispose or provoke DH including traumatic or premature occlusion, malocclusion, bruxism, acidic intake, medications, gastroesophageal reflux, stress, and saliva quality (2, 5, 23).

**Table 1:** Etiological or predisposing factors for dentine hypersensitivity.

Tooth related causes	
Tooth wear: Attrition, abrasion, erosion	Any form of tooth wear, either physiologic or pathologic, causes an irreversible, non-traumatic loss of dental hard tissues, leading to exposed dentine, open dentinal tubules, and painful sensations in response to various stimuli (5, 23, 24)
Aggressive brushing and type of toothpaste	Aggressive or vigorous brushing leads to tooth wear and DH (25). The abrasion caused by toothbrushing is also influenced by the abrasivity of the toothpaste. The type of toothpaste plays a key role, some ingredients may offer erosion protection, while abrasive particles and other components can exacerbate tooth wear, particularly on the already eroded surface and impact the interaction between active ingredients and the eroded tooth surface (26, 27)
Orthodontic treatment	DH is more prevalent in patients after orthodontic treatment than in the general population (28). Orthodontic treatment is positively associated with the prevalence of gingival recession and DH (29, 30). However, careful treatment can effectively prevent it (31). Moreover, orthodontic correction of the root toward the center of the alveolar envelope can reduce gingival recession risk (32)
Supporting tissue related causes	
Gingival recession, and periodontal disease	Recession of gingiva either by chronic trauma from tooth brushing etc., or due to periodontal inflammation may cause dentine exposure and DH symptoms (23, 33)
Oral hygiene behaviors	Abnormal oral hygiene behaviors like abrasive brushing, use of highly abrasive toothpaste, excessive brushing times, duration, and pressure results in gingival recession, dentine exposure and eventually lead to DH (23, 34)
Predisposing factors	
Traumatic occlusion/premature occlusion or malocclusion	Occlusal factors play an important role in development of non-carious cervical lesions, abfractions or gingival recession and hence may lead to DH (23, 35, 36)
Bruxism or parafunctional habits	Bruxism or parafunctional habits may predispose individual to tooth wear and hence DH (5)
Acidic intake	Exposure of teeth to extrinsic acids by excessive intake of acidic foods plays a causative role in erosive tooth wear and subsequent DH (23, 37)
Drugs	Medicines like aspirin, non-encapsulated HCL replacement, Vitamin C tablets, iron tablets, etc. can be sources of extrinsic acids that contribute to tooth wear and DH (23)
Reflux	Reflux or regurgitated acid from the stomach into the oral cavity, due to vomiting or gastroesophageal reflux, is the common intrinsic cause of erosive tooth wear, resulting in DH symptoms (23, 38, 39)
Stress	Emotional or mental stress can trigger parafunctional habits such as bruxism, clenching or grinding of teeth (40, 41). It may also induce gastroesophageal reflux, which can contribute to erosive tooth wear and DH symptoms (42)
Nature/quality of saliva	Salivary factors like flow rate, pH, buffering or neutralizing capacity, clearance, and composition play an imperative role in the development and progression of tooth wear and, consequently, DH (37, 43)

DH, dentine hypersensitivity.

#### 4.1.3 Clinical features and definition

DH is a chronic or long-lasting condition, subjective in nature, characterized by acute episodes of brief and intense pain in teeth that occurs from exposed dentine (3, 44). This pain is triggered by different external and non-noxious or non-harmful stimuli (44). It generally occurs in otherwise healthy teeth wherein the pain cannot be attributed to any other dental condition and may negatively affect QoL (44).

In 2002, the Canadian Advisory Board on DH updated the initial definition of DH by Holland GR, 1997 as “short, sharp pain arising from exposed dentine in response to stimuli typically thermal, evaporative, tactile, osmotic or chemical and which cannot be ascribed to any other form of dental defect or disease” (2).

After thorough consideration of the literature and an in-depth discussion, the panel recommended adding a few elements to the Canadian Advisory Board definition. The final agreed-upon definition was “a short, sharp, acute pain, subjective in nature arising from exposed dentine in otherwise healthy teeth in response to non-noxious stimuli, typically thermal, evaporative, tactile, osmotic or chemical and which cannot be ascribed to any other form of dental defect or disease.” This definition not only challenges clinicians to consider other potential causes of pain associated with tooth sensitivity, but also emphasizes the importance of differential diagnosis as well as, acknowledges individual differences in pain response to DH.

#### 4.1.4 Mechanisms underlying DH

The panel agreed that the most widely accepted mechanism of DH is the hydrodynamic theory proposed by Brännström (2, 5, 23). According to this theory, fluid flow within the dentinal tubules near the exposed surface increases or changes direction due to thermal, tactile, or osmotic stimuli. This alteration stimulates the baroreceptors or A-delta fibers around the odontoblasts, resulting in neural discharge. For DH to occur, the dentinal tubules must be open at both the surface and within the pulp (2, 5, 23). Some argued that this theory does not explain sensitivity in endodontically treated teeth. In such cases, the odontoblast receptor (OR) theory may apply. This theory suggests that exposed odontoblastic processes on the dentine surface are stimulated by various stimuli, leading to the release of neurotransmitters toward the nerve endings (23, 45).

#### 4.1.5 Barriers of DH diagnosis

One of the main barriers in DH identified by the panel was the lack of communication between patients and their dental practitioners (46). DH often is not perceived as a serious condition. Some individuals consider DH to be an inevitable aspect of aging leading them to develop coping behaviors rather than self-reporting their symptoms (47, 48). Others may endure the sensitivity but choose not to discuss it, either due to anxiety or because they prioritize other dental concerns that they may perceive as more serious (46). Moreover, many patients remain unaware that DH is both treatable and preventable (49), while many professionals also exhibit gaps in their knowledge of its prevention, diagnosis, and management (50).

The panel suggested that clinicians initiate conversations about the impact of DH on QoL to help patients better relate to the condition. Cost and time are additional barriers (46, 51). In busy dental practices, clinicians have limited time during consultations (46). In some countries, where the majority of the population relies on the public dental facilities, patients often present with severe dental conditions alongside DH. However, DH is not typically addressed in treatment plans, as clinicians are focused on more invasive dental procedures (46, 52).

The panel emphasized the need to involve other healthcare professionals such as dental hygienists in the consultation and diagnosis of DH as a mean to reduce the cost barrier. Additionally, the panel highlighted the lack of formal training on DH diagnosis and consequently the inadequate clinical skills of the clinicians, as additional factors that contribute to underdiagnosis of DH (14). Studies in the MEA region are needed to specifically identify the barriers to DH diagnosis and suggest more effective methods of patient-clinical communication to address the reported high prevalence of DH in the region (14).

### 4.2 Early, periodic, and comprehensive screening

#### 4.2.1 Screening

Many patients with DH may not self-report their condition, leading to overlooked early detection and prevention. The panel advised that screening for DH should be performed for all patients visiting dental clinics (2, 23). Considering the limited time during consultations, the panel suggested asking one screening question, such as “do you have sensitive teeth?” Clinicians can then proceed based on the patient's response. The panel also recommended pre-consultation aid, such as questionnaires that the patient can respond to while waiting to see his/her dentist, or before they arrive for their appointment (53). These responses can be reviewed by the clinician to maximize the use of appointment time. This approach could be economically beneficial in countries where the majority of patients visit public oral health facilities. Additionally, the panel also emphasized the importance of patient education as a mean to prevent the occurrence of DH or reduce its burden on affected patients. Equally important is ensuring that dental professionals are adequately trained to recognize and diagnose DH. Several studies have shown that knowledge gaps among clinicians, can limit accurate diagnosis and management of DH. Investigations outside the MEA region (50, 54, 55) and within MEA countries (14, 56) highlight that both undergraduate training and continuing education are essential to improve diagnostic consistency. Thus, alongside patient education, strengthening professional training is critical to ensure early and reliable detection of DH.

#### 4.2.2 History taking

Comprehensive clinical history taking is crucial in patients complaining of DH-related pain. The panel suggested that clinicians should inquire about the type and duration of pain, its triggering factors, relieving factors, and any prior treatments the patient may have received (2, 23). Moreover, a comprehensive medical history taking is necessary to accurately identify DH underlying risk factors. For example, inhalation therapies for asthma can reduce salivary buffering capacity and flow, thereby leading to increased erosive wear and consequently DH (57). Clinicians should also consider the patient's dietary habits (e.g., frequent intake of acidic foods and beverages such as carbonated drinks, citrus fruits, and vinegar-containing products), lifestyle factors (e.g., stress levels that may contribute to bruxism or clenching, smoking, alcohol consumption), and oral health behaviors (e.g., aggressive or improper toothbrushing techniques, frequency and duration of brushing, use of abrasive whitening toothpastes, or overuse of dental floss or interdental aids). Each of these factors can predispose to enamel/dentine wear or gingival recession and thereby contribute to DH (2, 23).

DH episodes are often short-lived, and patients may develop coping behaviors, such as, avoiding cold foods or eating and drinking from the opposite side of the mouth (58). The unpleasant symptoms of DH impacts QoL, making QoL assessment essential in clinical practice, as it can influence the treatment plan (4, 59). The Dentine Hypersensitivity Experience Questionnaire (DHEQ) is a widely accepted and validated tool that evaluates the subjective impact of DH in five areas of life: functional restrictions (e.g., slower eating), coping behaviors (e.g., warming food and drinks), emotional impact (e.g., annoyance), social impact (e.g., difficulty conversing), and personal identity impacts (e.g., feeling old) (60). While the original version of DHEQ has 48 items and can be time consuming limiting its clinical utility, a shorter 15-item version (DHEQ-15) was developed for use in general dental practices (60). However, the panel raised concern about its applicability in routine consultations due to time constraints. Consequently, there is a recognized need to further simplify DHEQ to enhance its utility in everyday clinical practice.

#### 4.2.3 Clinical examination and exclusion of other conditions

To properly diagnose DH, it is essential to exclude other conditions that cause similar pain, e.g., cracked tooth, dental caries, root resorption, defective or fractured restorations, post-operative sensitivity (from restorative, periodontal and bleaching procedures), dental trauma, occlusal trauma, cervical plaque, gingivitis, periodontal disease, marginal leakage, pulpitis, enamel or dentine hypoplasia, hypomineralization and other pathologic conditions like cysts (2, 13, 23, 52). In addition to visual and tactile examination, various diagnostic methods can be employed to exclude other conditions, including percussion, palpation, vitality test, radiographic examination, and transillumination (2, 13, 23, 52).

#### 4.2.4 Diagnostic measures

Owing to its subjective nature, quantifying DH, and its impact on patients in clinical setting can be challenging. Therefore, clinicians must rely on the patient's self-report of pain. Stimulus-based diagnostic tests are valuable for diagnosing DH (2, 13, 23, 52). The panel recommended starting with a simple mechanical or tactile stimulus test. This involves using a sharp explorer or conventional or pressure-sensitive dental probe to gently move over the exposed dentine in a mesio-distal direction while observing the patients' response (2, 13, 23, 52).

As a next step, the panel recommended performing a cold air blast test using three-way syringe, which is commonly used by clinicians and in clinical studies (1, 14). The panel agreed that Schiff scoring, which is response-based assessment, requires minimum time to implement clinically. The panel also recommended using a numerical rating scale, where patients can self-assess DH pain from 0 to 10, in which 0 would indicate no pain and 10 indicates severe pain. The Schiff scale has been reported to have high specificity (91%), while the numerical scale has high sensitivity (81.9%) (61). The numerical rating can be particularly useful for assessing treatment progress and follow-up; a lower rating during follow-up visits would indicate an improvement in DH symptoms.

The panel also suggested exploring the aid of clinical photographs, intraoral scanners and AI based intraoral scanning software in future for DH diagnosis (62). These technologies may serve as efficient pre-screening tools that can also save time (62).

To assess and record DH, the panel suggested documenting the severity, location (localized or generalized) and whether it is primary (no previous treatment) or secondary (prior treatment). The findings should be recorded clearly so that other clinicians can easily understand and localize the problem. Imperial charting systems, such as periodontal charting for mobility and furcation involvement, could be adapted for recording DH (63). Currently, there is no objective classification system for DH severity analogous to those available for gingival recession, thus clinicians must rely on their judgment to classify or quantify DH. Manual or electronic health record systems, like Axium or Doc 32, which offer customization, can be utilized (64). These systems should be adaptable to the clinical setting and include an editable section for clinicians to write recommendations or treatment notes. Proper documentation of DH findings allows for comparison between initial and subsequent visits, facilitating the monitoring of treatment progress.

### 4.3 Optimizing DH management with a simplified algorithm is essential

**Figure 2:**
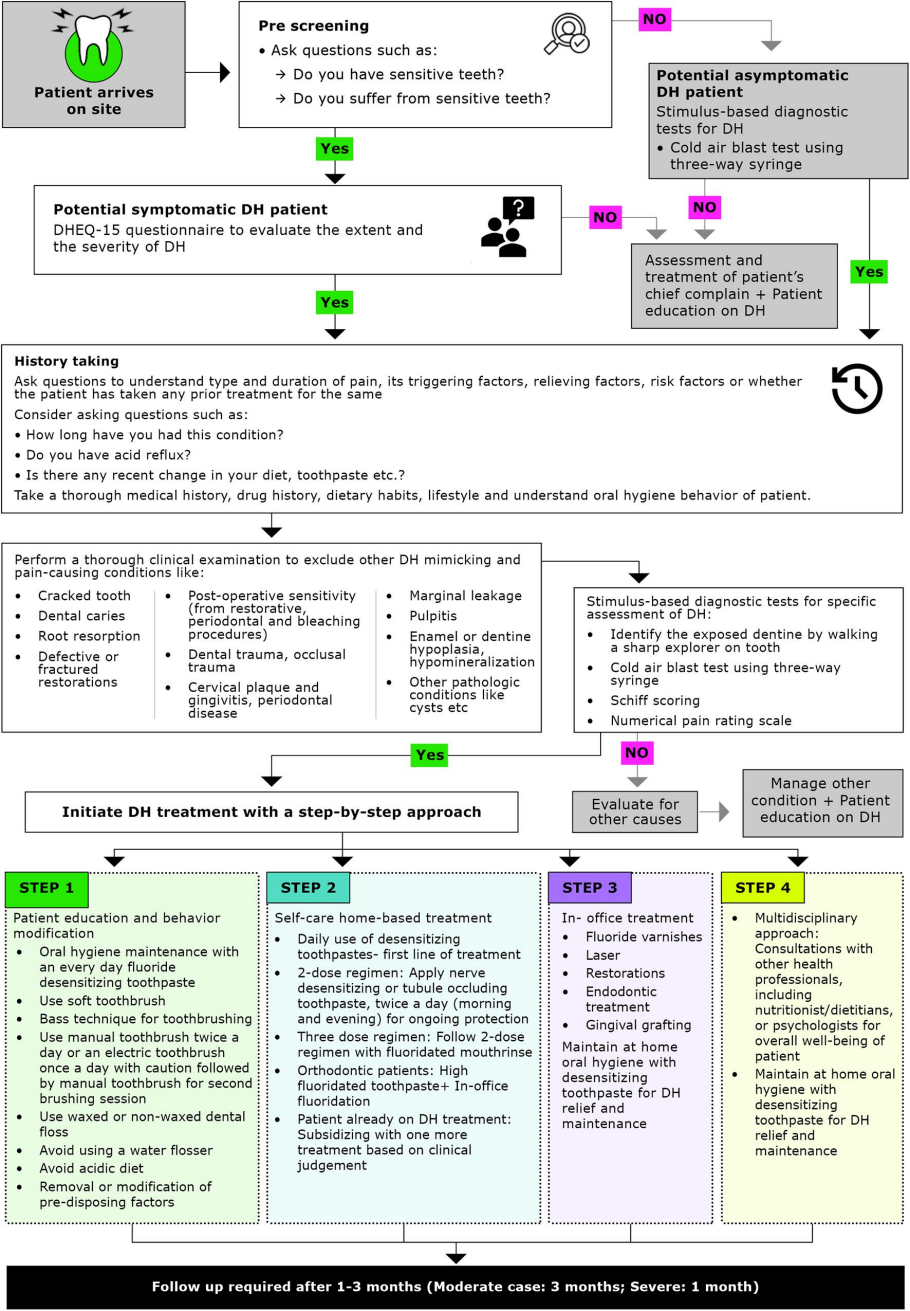
Simplified algorithm for management of dentine hypersensitivity. DH, dentine hypersensitivity; DHEQ, dentine hypersensitivity experience questionnaire.

Treatment for DH should follow a step-by-step approach, beginning with simple strategies such as patient education and behavior modification, followed by self-care home-based treatments, and progressing to more complex in-office treatment as needed (13, 23). The complexity as well as the invasiveness of treatment strategies should be chosen based on the severity or extent of DH. The Canadian Advisory Board on DH has previously recommended reversible procedures to be employed before nonreversible ones depending on the severity and extent of each condition (2). A simplified algorithm has been developed to provide evidence-based practical guidance for clinicians (Figure 2). Clinicians should consider the following approaches for the prevention and management of DH (Table 2).

**Table 2:** Evidence-based recommendations from experts from Middle East and Africa.

Expert opinions	Published evidence	Evidence grade and level
Screening and diagnosis		
Screening is crucial for the diagnosis of DH. All patients visiting dental clinics must be screened for DH. Accordingly, clinicians should be well trained	Canadian advisory board also recommend that screening is essential for identifying DH (2)	C
It is important for clinicians to identify patient- specific risk factors. Predisposing factors including diets, drugs, acid reflux (dietary or systemic) and importantly saliva quality should be considered while evaluating the DH patients	DH in is associated with factors related to dental erosion like clinical, dietary, and salivary variables (37). Endogenous acid from acid reflux or exogenous or dietary acids cause erosion and subsequently DH (38, 39, 65)	BIII
Schiff scale or numerical pain scale should be used clinically as a diagnostic measure for DH	The accuracy of different diagnostic measures for DH ranged from 0.729 for Schiff scale to 0.750 numerical pain scale. The highest sensitivity value was 81.9% for numerical pain scale. The Schiff scale presented the highest specificity (91%). The visual analog, numerical, verbal evaluation, faces pain, and Schiff scales were accurate for DH diagnosis. The Schiff scale was the preferred scale for DH assessment (66)	BIII
Management		
Use of desensitizing agents as first line treatment, more invasive treatment are recommended when needed in severe cases. As first line of treatment, DH patients should be prescribed nerve desensitizing and/or tubule occluding dentifrices. Newer toothpastes with both nerve desensitizers and tubule occluding agent can also be considered	Studies have shown efficacy for both nerve desensitizing agents (for example 5% potassium nitrate toothpaste) and tubule occlusion ingredients (for example strontium, stannous fluoride, arginine and calcium sodium phosphosilicate and potassium-based toothpastes in relieving symptoms of DH (67–70). Limited studies have also shown effect of toothpastes containing combination of stannous fluoride and potassium nitrate in reducing DH (71–73). RCTs have shown toothpaste containing combination of potassium nitrate, and strontium chloride was effective against DH after 3 days, 4 and 8 weeks (74, 75)	GPP, AI
Fluoride-based mouthwash can be used after desensitizing dentifrice for DH	Fluoridated formulations including mouthwash, toothpaste, solution, gel, and varnish relieves DH symptoms (76, 77). Literature evidence suggests self-administered agents including fluoridated mouthwash is effective in managing DH (78, 79)	GPP, AI
Electric toothbrushes should be used with caution in DH patients and those with thin gingival phenotype due to risk of increasing DH. If required, newer pressure sensitive electric toothbrushes can be considered	A 35%–40% reduction in DH related pain as compared to baseline was observed with use of powered or electric toothbrush (80)	GPP, AI
Follow up should be done at an interval of 1–3 months (Moderate case: 3 months; Severe: 1 month)	NA	GPP
For moderate cases topical desensitizers fluoride varnish is preferred in-office treatment. Other treatments include composite filling, GIC, or grafting etc.	Fluoride varnish such as Duraphat and Gluma have showed significant reduction in DH and VAS score at 5 min and 7 days with no superiority (81). Recommendations from Asian geographies suggest in-office treatment for DH includes composite filling, GIC, or grafting etc. (23).	C
Erbium and chromium laser irradiation in combination with topical treatment can also be considered. Nd: YAG laser and the diode laser also helps managing DH effectively	Literature suggests that laser therapy including Nd: YAG laser and the diode laser (high and low power) was found to be effective in the treatment of DH. However, the level of effectiveness depends on the laser used (82, 83)	AI
Invasive treatment (root canal treatment) is considered in severe cases	Recommendations from Asian geographies suggest endodontic therapy as last resort for treating DH (23)	C
Education		
Awareness and education on DH is needed for both dental professionals and public. Continuing education and clinical training for dentists is needed. Undergraduate curricula in dental schools in the region should include diagnosis and treatment of DH and assessments of attainment of learning outcomes	In Nigeria, knowledge gaps regarding diagnosing and managing DH were observed among practicing dentists. On evaluation of 36 knowledge-based questions, only 1.8% dentist provided the correct responses for at least 25 questions (14). In Saudi Arabia, 66.6% dental students did not know regarding the steps to diagnose DH. Students lacked the knowledge and confidence to manage DH in clinics (84). In Brazil, 36.7% and 18.6% of the people were unaware that DH can be both prevented and treated, respectively (49)	BIII
Research		
Additional large-scale studies are recommended on specific risk factors and their impact on the observed prevalence of DH. Results obtained from these studies can be used to design randomized clinical trials to assess the most effective approaches to reduce the burden of DH in the region	NA	GPP

AI: evidence obtained from a single randomized controlled trial (RCT) or a meta-analysis of RCTs; BIII: evidence obtained from well-designed nonexperimental descriptive studies, such as comparative studies, correlation studies, and case studies; C: evidence obtained from expert committee reports or opinions and/or clinical experiences of respected authorities; DH, dentine hypersensitivity; NA, not applicable or not available; GPP, good practice point.

#### 4.3.1 Patient education and behavior modification

A dedicated counseling session for patient education and behavior modification is essential for the prevention and management of DH. The panel recommended educating patients on behavioral modifications, focusing on the following areas:

##### 4.3.1.1 Oral hygiene maintenance and toothbrushing

The panel emphasized the importance of maintaining good oral hygiene and suggested training patients on proper brushing techniques, including how to position the brush, apply pressure, and effectively clean their teeth. The Bass technique was agreed as the correct method for brushing, as horizontal brushing may lead to increased gingival recession, cervical abrasions and exacerbate DH (85). It is advised that patients use a soft-bristled toothbrush and avoid highly abrasive teeth whitening toothpaste, as these products often contain harsh, coarse granules that can increase DH (2, 23). Regular dental check-ups and monitoring of plaque indices are also essential for effective management (2, 23).

##### 4.3.1.2 Electric toothbrush

The panel emphasized that when recommending an electric toothbrush, it is important to advise the patient to use it gently and passively, without applying additional pressure as is done with manual toothbrushing (86, 87). The brush tip is designed to do the work, and any additional pressure can lead to gingival recession. An electric toothbrush should be used cautiously in people with DH or a thin gingival phenotype, as additional pressure may aggravate DH or cause gingival recession. The use of a pressure-sensitive toothbrush can help control the amount of pressure applied (88). The panel prefers that patients with DH use an electric toothbrush once daily to help minimize brushing pressure. For the second daily brushing, patients may use a manual toothbrush, or alternatively, use a manual toothbrush for both brushing sessions.

##### 4.3.1.3 Interdental aid

The panel advised against the use of water flossers in people with DH, as the water spray can act as a thermal stimulus, triggering DH pain. Instead, they recommended the use of waxed or non-waxed floss (23).

##### 4.3.1.4 Dietary habits

It is essential to educate patients on avoiding acidic foods and beverages such as fizzy drinks, acidic beverages, lemon juice with vinegar etc. (2, 23). Clinicians should advise patients to limit frequent intake of highly acidic, low-mineral foods and drinks that increase erosive wear and may exacerbate DH, for example, carbonated soft drinks (including diet sodas), energy/sports drinks, fruit juices and citrus fruits, sour candies/lozenges, vinegar-based foods, and frequent sipping of acidic beverages. In contrast, calcium/phosphate-rich foods (milk, cheese, plain yogurt) are less erosive and can aid remineralization (23, 74–89).

When complete avoidance is impractical, recommend harm-reduction measures: drink acidic beverages with meals, use a straw, avoid swishing, rinse with water or a fluoride mouthwash after exposure, chew sugar-free gum to stimulate saliva, consume a calcium-rich snack afterward, and delay toothbrushing for 30–60 min after acidic intake. These strategies help balance nutritional benefits (e.g., fruit consumption) with enamel protection (2, 23).

##### 4.3.1.5 Removal of predisposing factors

Selective occlusal correction to resolve premature contacts should be considered with caution in cases of trauma from occlusion or premature contacts. For individuals with bruxism or other parafunctional habits, the use of an occlusal splint night guard is recommended. Any other predisposing factor identified during history taking and clinical evaluation should be addressed, removed, or modified as necessary (2, 23).

#### 4.3.2 Self-care home-based treatment

**Figure 3:**
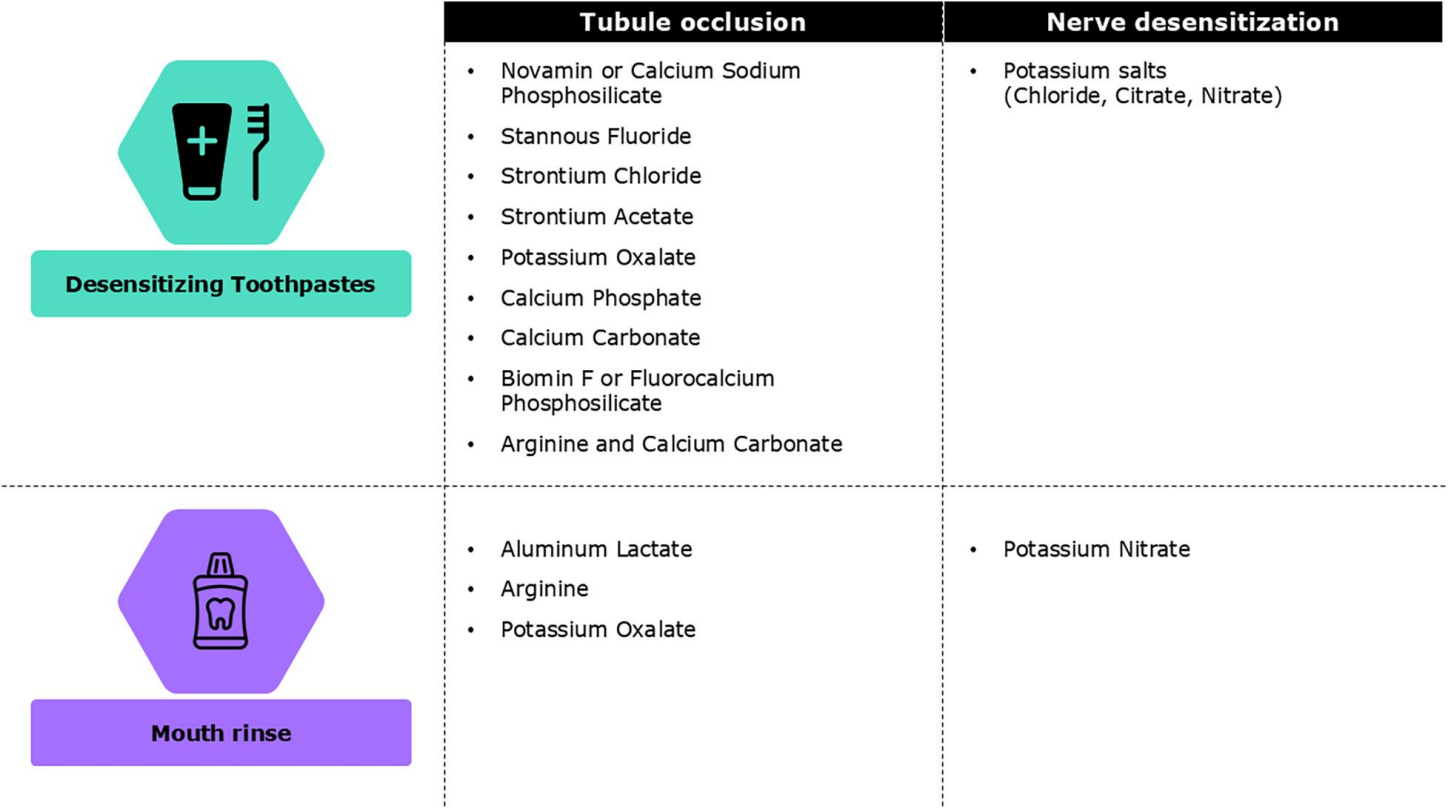
Different types of self-care home based treatments for dentine hypersensitivity (23).

Desensitizing agents are the most widely used among all available self-care home-based regimes (Figure 3). The daily use of desensitizing toothpastes is a non-invasive, inexpensive, and effective first-line treatment for DH. Desensitizers with tubule-occluding agents, such as strontium acetate, stannous fluoride, and more recently calcium sodium phosphosilicate, reduce fluid flow across the dentinal tubules by blocking them while nerve desensitizers containing potassium salts suppress nerve stimulation associated with intratubular fluid movements (90). Toothpastes containing stannous chloride can also reduce DH and improve oral health-related QoL (91, 92). However, no clinical studies have assessed the desensitizing effect of this formulation (91, 93, 94). Desensitizing toothpaste can be applied with a toothbrush or directly on sensitive areas using a fingertip before brushing (95). Patients should be advised to continue using desensitizing toothpaste even after symptoms have subsidized. Discontinuing may result in the recurrence of DH, possibly due to the gradual reopening of dentine tubules after stopping the use of desensitizing toothpaste (96). For patients undergoing orthodontic treatment, high-fluoridated toothpaste, along with in-office fluoridation should be considered.

If the patient is already undergoing treatment for DH, clinical judgement should guide whether to continue the current treatment or adjust it. Adding a fluoride mouthwash or considering an in-office treatment could be beneficial (23).

#### 4.3.3 In- office treatment

In cases where there is no improvement in DH symptoms despite self-care home-based treatments, or if the symptoms are worsening, in-office treatments may be necessary. These can include the professional application of fluoride varnishes, topical desensitizing agents that contain glutaraldehyde and hydroxyethyl methacrylate (HEMA), laser therapy, and restorative interventions when indicated. Highly-fluoridated toothpastes are typically prescription home products and should not be listed as in-office materials. Fluoride varnishes and glutaraldehyde/HEMA products have been widely reported as effective in clinical studies (97–81). Clinicians should note that some in-office desensitizing agents may affect the bond strength of subsequently placed resin composites; when adhesive restorations are planned, follow manufacturer recommendations, consider delaying bonding procedures, or perform appropriate surface management to minimise bonding interference (98). In cases of extreme sensitivity, local anaesthesia may be used to apply desensitizing materials. For severe cases, endodontic treatment or extraction may be considered. If gingival recession is present, any existing root caries should be treated first. In cases with aesthetic concerns, gingival grafting may be considered (2, 13, 23, 52, 81).

#### 4.3.4 Multidisciplinary approach

To promote the overall wellbeing of the patient, consultations with other health professionals, including nutritionist/dietitians, or psychologists (to address stress, which is one of the causative factors of DH) must be considered.

#### 4.3.5 Follow-up

Regular follow-up for DH patients is essential for achieving better patient outcomes. Follow-up must be scheduled every 2–3 months. However, in severe cases, monthly follow-ups may be necessary. It is not mandatory for a patient to visit the clinic in person for every follow-up; this can also be managed through telephonic interviews conducted by dental hygienists or assistants.

### 4.4 Raising DH awareness among dentists and the public

#### 4.4.1 Education for dental professionals

The panel highlighted concerns regarding the education, awareness, and clinical application of DH among dental students (Table 2). They emphasized that DH should be integrated into the curricula of public health, endodontic, and periodontal specialties, adopting an interdisciplinary approach. It was suggested that DH be considered an essential component of dental pedagogy.

Tutorial-based education on DH alone is insufficient, its importance and clinical application should be conveyed through problem-based learning. As clinical education typically begins later in the curriculum, DH should be introduced to students in their later years. Implementing a competency-based approach to DH education is recommended. Both dental hygienists and clinicians should be trained in managing DH. Scientific organizations could play a crucial role by discussing DH at national and international conferences or symposiums to raise awareness and advance knowledge.

#### 4.4.2 Education for public

The panel discussed the importance of public education and awareness in the prevention and management of DH (Table 2). Increased awareness can lead to greater seriousness about the condition, which in turn raises the demand for DH management. It is crucial to advertise on social media with clear messages that DH treatment is effective, rather than merely discounting the symptoms. The panel also suggested that dental clinics should display anatomical charts to raise awareness about healthy gums and teeth, as well as DH in relation to gingival recession. Educational materials should be made available on dental clinics' websites or through health associations to further public awareness.

### 4.5 Limitations

The expert panel included representatives from eight MEA countries, the literature cited in this review is predominantly derived from studies conducted outside the MEA region. The limited availability of region-specific data across many MEA contexts presents a broader challenge. While prevalence figure from countries such as Nigeria, Saudi Arabia, and Oman are included, some MEA nations remain underrepresented. We acknowledge that the consensus-based recommendations may not fully capture the diversity and unique characteristics of all regional populations. This limitation underscores the need for more locally driven research to strengthen the evidence base and ensure future guidelines are more representative of the entire MEA region.

## 5 Conclusion

DH is an increasingly prevalent clinical condition that affects QoL. The expert panel's recommendations provide a structured, evidence-based framework for diagnosing and managing DH in the MEA region. By offering a simplified diagnostic and treatment algorithm, the guidelines address the unique factors contributing to DH, such as dietary habits, oral behaviors.

The expert panel underscores the importance of identifying underlying predisposing or etiologic factors to ensure accurate diagnosis and effective management. Given the subjective nature of DH and the tendency for symptoms to be underreported, routine screening all dental patients is recommended. A comprehensive clinical history and thorough clinical examination are vital to differentiate DH from other conditions with similar presentations. Stimulus-based diagnostic tests, along with clear documentation of key findings (e.g., location and severity), support accurate diagnosis and facilitate appropriate follow-up.

Management of DH should follow a stepwise approach, beginning with the elimination of modifiable risk factors and the promotion of optimal oral hygiene practices. First-line treatment should include home-based self-care strategies such as twice-daily use of desensitizing toothpaste (tubule occluding or nerve desensitizers) and/or fluoridated mouthwash. For more persistent or severe cases, escalation to in-office interventions may be necessary. A multidisciplinary approach, engaging dental specialists, nutritionists, and other healthcare professionals can support holistic patient care, addressing both oral and systemic contributors to DH.

Overall, the panel emphasizes the importance of early diagnosis, preventive education, and tailored personalized interventions to ensure effective DH management. Integration of DH awareness into both public health initiatives and professional dental education is crucial to reducing DH prevalence and improving QoL for affected individuals across the MEA region.
