# Supplementary material for: Evidence-based recommendations for diagnosing and managing dentine hypersensitivity in clinical practice: insights from the Middle East and Africa
Source: Front Oral Health. 2025 Nov 7;6:1663984. doi: 10.3389/froh.2025.1663984 (PMC12634618; doi:10.3389/froh.2025.1663984)
Supplement: Supplementary file 1 [file Datasheet1.docx]

Supplementary materials

# 1 Supplementary Tables

**Table 1. List of countries in Middle East and Africa**

| **Countries in Middle East and Africa region** | | | | | |
| --- | --- | --- | --- | --- | --- |
| Turkey | Bahrain | Ghana | Chad | Republic of the Congo | Réunion |
| Iraq | Iran | Mozambique | Guinea | Liberia | Comoros |
| Saudi Arabia | Nigeria | Ivory Coast | Tunisia | Mauritania | Western Sahara |
| Yemen | Ethiopia | Madagascar | Rwanda | Gabon | Cape Verde |
| Syria | Egypt | Angola | South Sudan | Namibia | Mayotte |
| United Arab Emirates | Democratic Republic of the Congo | Cameroon | Benin | Botswana | São Tomé and Príncipe |
| Israel | South Africa | Nigeria | Somalia | Lesotho | Seychelles |
| Jordan | Tanzania | Burkina Faso | Burundi | Equatorial Guinea | Pakistan |
| Palestine | Kenya | Mali | Togo | Gambia |  |
| Lebanon | Algeria | Malawi | Libya | Guinea-Bissau |  |
| Oman | Uganda | Zambia | Sierra Leone | Mauritius |  |
| Kuwait | Sudan | Senegal | Central African Republic | Swaziland |  |
| Qatar | Morocco | Zimbabwe | Eritrea | Djibouti |  |

The expert panel comprised of 12 experts from 8 countries in Middle East and Africa region (highlighted above).

**Table 2. Recommendation grading structure and hierarchy of evidence^1^**

| **Level** | **Type of evidence** | **Grade** | **Evidence** |
| --- | --- | --- | --- |
| I | Evidence obtained from a single randomized controlled trial (RCT) or a meta-analysis of RCTs | A | At least one RCT as part of a body of literature of overall good quality and consistency addressing the specific opinion (evidence level I) without extrapolation |
| IIa | Evidence obtained from at least one well-designed controlled study without randomization | B | Well-conducted clinical studies but no randomized clinical trials on the topic of opinion (evidence levels II or III); or extrapolated from level-I evidence |
| IIb | Evidence obtained from at least one other well-designed quasi-experimental study |  |  |
| III | Evidence obtained from well-designed nonexperimental descriptive studies, such as comparative studies, correlation studies, and case studies |  |  |
| IV | Evidence obtained from expert committee reports or opinions and/or clinical experiences of respected authorities | C | Expert committee reports or opinions and/or clinical experiences of respected authorities (evidence level IV). This grading indicates that directly applicable clinical studies of good quality are absent or not readily available |
|  |  | GPP | Recommended GPPs based on the clinical experience of the experts |

# 2 Supplementary Figure

**Figure 1. Search and selection process of articles**


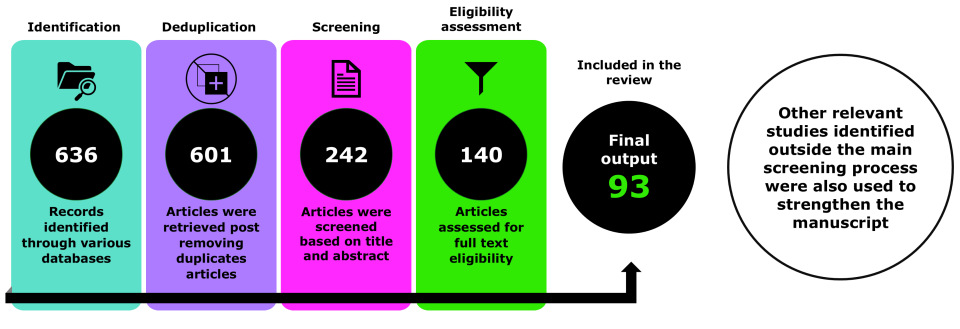


# 3 Reference

1. National Collaborating Centre for Mental Health (UK). Self-Harm: The Short-Term Physical and Psychological Management and Secondary Prevention of Self-Harm in Primary and Secondary Care. Leicester (UK): British Psychological Society (UK) (2004) National Institute for Health and Care Excellence: Guidelines.
